# Systematic review of probiotics as an adjuvant treatment for psychiatric disorders

**DOI:** 10.3389/fnbeh.2023.1111349

**Published:** 2023-02-09

**Authors:** Evan Forth, Benjamin Buehner, Ana Storer, Cassandra Sgarbossa, Roumen Milev, Arthi Chinna Meyyappan

**Affiliations:** ^1^Department of Psychiatry, Providence Care Hospital, Kingston, ON, Canada; ^2^Centre for Neuroscience Studies, Queen’s University, Kingston, ON, Canada; ^3^Department of Psychiatry, Queen’s University, Kingston, ON, Canada; ^4^Department of Psychology, Queen’s University, Kingston, ON, Canada

**Keywords:** probiotics, psychiatric illness, psychotropics, adjuvant therapy, gut-brain-axis probiotics, gut-brain-axis, Major Depressive Disorder

## Abstract

**Introduction:**

Many psychiatric illnesses have been linked to the gut microbiome, with supplements such as probiotics showing some efficacy in alleviating the symptoms of some psychiatric illnesses. The aim of this review is to evaluate the current literature investigating the effects of adjuvant probiotic or synbiotic administration in combination with first-line treatments for psychiatric illnesses.

**Method:**

A systematic search of four databases was conducted using key terms related to treatments for psychiatric illnesses, the gut microbiome, and probiotics. All results were then evaluated based on specific eligibility criteria.

**Results:**

Eight studies met eligibility criteria and were analyzed for reported changes in outcome measures used to assess the symptoms of psychiatric illness and the tolerability of treatment. All Major Depressive Disorder (MDD) (*n* = 5) and Generalized Anxiety Disorder (GAD) (*n* = 1) studies found adjuvant probiotic or synbiotic treatment to be more efficacious in improving the symptoms of psychiatric illness than the first-line treatment alone or with placebo. The schizophrenia studies (*n* = 2) found adjuvant probiotic treatment to have no significant difference in clinical outcomes, but it was found to improve the tolerability of first-line antipsychotics.

**Discussion and conclusion:**

The findings of the studies included in this review suggest the use of adjuvant probiotic treatment with selective serotonin reuptake inhibitors (SSRIs) for MDD and GAD to be superior to SSRI treatment alone. Probiotic adjuvant treatment with antipsychotics could be beneficial for improving the tolerability of the antipsychotics, but these findings do not suggest that adjuvant probiotic treatment would result in improved clinical outcomes for symptoms of schizophrenia.

## Introduction

The human gastrointestinal (GI) tract houses trillions of microorganisms, which have co-evolved with their host and collectively contain over 100 times as many genes as the human genome (Bermon et al., 2015). Colonization of the gut begins at birth, being influenced by the mode of delivery and breastfeeding (Martin et al., 2016) and through the gut’s microbial composition somewhat stabilizes throughout adulthood, factors such as the environment, diet, medication, genetics, and age continue to shape microbiota composition and function throughout one’s life (Li et al., 2014; Heiman and Greenway, 2016; Odamaki et al., 2016; Cussotto et al., 2019). There is a well-established, bidirectional connection between the gut microbiome and the brain, known as the gut-brain-axis (GBA). This communication includes portions of the sympathetic and the parasympathetic nervous system, the enteric nervous system, as well as both neuroimmune and neuroendocrine signaling (Cryan et al., 2019; Morais et al., 2021; Liu et al., 2022). Research suggests that the GBA may influence a variety of neurological functions, including the pathology of psychiatric disorders.

Major Depressive Disorder (MDD), Generalized Anxiety Disorder (GAD) and Schizophrenia (SZ) are widely known and severe psychiatric disorders. MDD is characterized by pervasive depressed mood and/or loss of interest or pleasure, along with an array of other possible psychiatric and physiological symptoms, and is the leading cause of disability worldwide (Evans-Lacko et al., 2018). GAD has a similarly significant impairment in daily functioning (American Psychiatric Association [APA], 2013), and is characterized by a persistent, exaggerated worry about everyday events. MDD and GAD are also somewhat gendered illnesses, with the prevalence in women being reported as 1.5 to 3 times that of men (Vesga-López et al., 2008; Sabic et al., 2021). Antidepressant medications such as selective serotonin reuptake inhibitors (SSRIs) are considered a first-line therapy for MDD and GAD (Gelenberg et al., 2010; Baldwin et al., 2011). SZ is a highly heterogenous psychotic disorder, characterized by continuous or relapsing episodes of positive symptoms like delusions, hallucinations and irrational thoughts or actions, and negative symptoms like lethargy, apathy, and social withdrawal. Second-generation antipsychotics are considered the first-line treatment for SZ (Patel et al., 2014). Gender differences found in schizophrenia are less consistent, with some reports of equal prevalence between men and women, and some reports of increased prevalence among men (Ochoa et al., 2012).

Various studies have found the microbiota composition of patients with these psychiatric disorders to be significantly different from those of healthy controls (Zhu et al., 2020; Nikolova et al., 2021). Interestingly, fecal microbiota transplants from psychiatric patients to germ-free rodents have been shown to induce symptoms similar to those associated with the disorders of the donors (Bercik et al., 2011; Neufeld et al., 2011). Certain probiotics or fecal microbiota transplants from healthy patients have also helped alleviate symptoms and induced positive outcomes in patients with psychiatric disorders (Meyyappan et al., 2020; Johnson et al., 2021). As such, there is emerging evidence to suggest that the gut microbiome and the GBA play a crucial role in inducing and modulating psychiatric disorders.

A significant subset of patients affected by these disorders are treatment-resistant or experience adverse effects when taking antidepressants or antipsychotics. Antipsychotic usage is commonly associated with adverse metabolic and endocrine effects such as weight gain and insulin resistance (De Hert et al., 2011), while SSRIs frequently induce unpleasant side effects such as nausea, insomnia, drowsiness and agitation (Hirsch and Birnbaum, 2017). Such adverse effects contribute to low treatment compliance and tolerability. Low compliance and inconsistent efficacy indicate that there is a need to explore alternative treatments, or approaches to counteract these unwanted side effects.

Interestingly, both antipsychotics and antidepressants have been found to have antimicrobial properties (Munoz-Bellido et al., 2000; Maier et al., 2018). It is thought that such psychotropic medications can modulate the gut microbiome, and consequently influence the GBA. Whether the therapeutic benefits or the adverse effects of these medications are influenced, in part, by their impact on the GBA remains to be determined, however, several recent studies have indicated that the gut microbiome composition could be used as a biomarker to predict pharmacological treatment outcomes (responders versus treatment resistance) in MDD and SZ (Fontana et al., 2020; Ciocan et al., 2021; Yuan et al., 2021). This suggests that the GBA could play a significant role in the efficacy and tolerability of psychotropic medication. This evidence, combined with the aforementioned therapeutic benefits of microbiome modulation on psychiatric disorders, and the proven ability of probiotics to normalize metabolic issues (Le Barz et al., 2015), suggest that combining psychotropic medication with gut microbiome targeting treatments could have beneficial results. The aim of this systematic review is to evaluate the current literature investigating the effect of adjuvant probiotic or synbiotic (a combination of probiotics and prebiotics) treatment on clinical outcomes and tolerability of first-line psychotropic treatments. We conducted this systematic review as a means to gather scientific evidence and provide a comprehensive and current overview of this topic.

## Methods

### Literature search strategy

**FIGURE 1:**
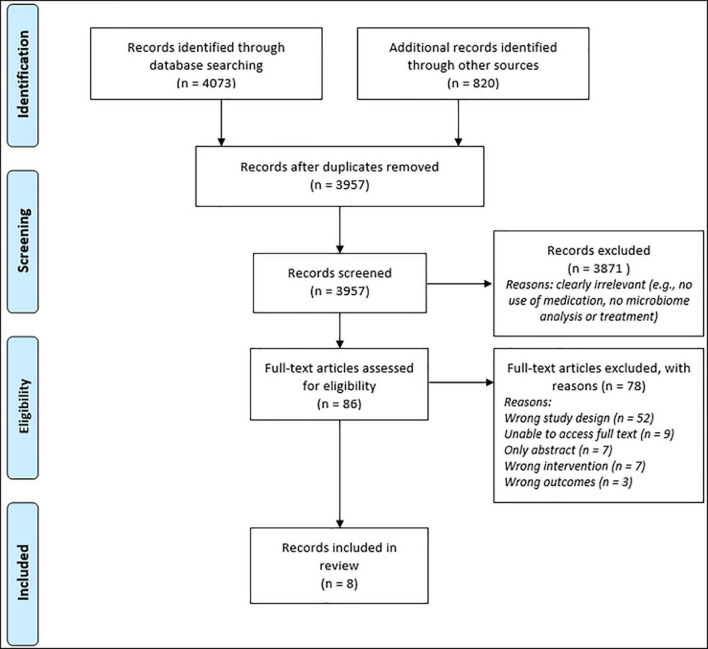
Flow chart showing literature search and screening process using PRISMA guidelines.

This review was carried out according to the Preferred Reporting Items for Systematic Reviews and Meta-Analyses (PRISMA) guidelines (Figure 1; Moher et al., 2009). A systematic search was conducted using 4 databases (MEDLINE, EMBASE, PsycINFO, and Web of Science) to identify relevant studies using the following search terms:(antidepressant OR selective serotonin reuptake inhibitors, SSRI OR SNRI OR TCA OR MAOI OR anti-anxiety drugs OR anxiolytics OR benzodiazepines OR beta-blockers OR antipsychotic medication OR mood stabilizer) AND (microbiome OR microbiota OR gut bacteria OR intestinal bacteria OR dysbiosis OR bacteriostatic OR bactericidal OR antibiotic OR bacterial therapy OR bacteriotherapy OR psychobiotic OR microbial therapy OR fecal microbiota transplant OR probiotic) AND (depression OR depressive disorder OR major depression OR bipolar OR mood disorders OR affective disorders OR stress, psychological OR anxiety OR anxiety disorder OR generalized anxiety disorder OR social anxiety disorder OR PTSD OR OCD OR mania OR panic OR phobia OR psychiatric illness). The database searches were supplemented by retrieval of any additional papers meeting eligibility criteria that were cited in reference lists of relevant review articles yielding 820 additional articles. Searches were conducted in January and February 2022 and yielded 3957 studies after duplicates were removed. Studies that were excluded during full-text screening were rejected due to wrong study design, including the article being a review article or abstract only, and wrong study outcomes. Articles rejected due to “wrong study outcome” did not measure changes in psychiatric symptoms in response to the use of a microbiome-targeted therapeutic as an adjuvant to medication for psychiatric illness.

### Eligibility criteria

Eligible articles were restricted to those that were published in peer-reviewed journals and were written in English. Studies eligible for inclusion involved clinical samples that assessed changes in psychiatric wellbeing after standard treatment indicated for psychiatric illness and an adjuvant therapeutic targeting the microbiome.

### Study selection

Two authors (BB and AS) completed the initial search of the databases, adhering to the search strategy as described above. Two authors (EF and one of BB or AS) independently assessed the titles and abstracts of records retrieved from a systematic search according to the identified inclusion and exclusion criteria. Two authors (BB and EF) completed the full-text review. Any disagreements were resolved by a fourth author (AC). Quality assessment of eligible articles was completed by a fifth author (CS).

### Study quality

Quality assessment of articles was completed using Covidence’s built-in, Cochrane Handbook for Systematic Reviews of Interventions, Risk of Bias (RoB) template. The Cochrane RoB tool assesses the risk of bias for the following domains: sequence generation, allocation concealment, blinding of participants and personnel, blinding of outcome assessment, incomplete outcome data, and “other” sources of bias. Most studies presented with a low level of bias. Studies where there was no mention of blinding to participants, personnel, outcome assessors, or allocation of treatment, were assigned a “high” judgment in risk of bias. In studies where there was no given description or details regarding one of the RoB domains (i.e., sequence generation), the risk of bias was assigned as “unsure.” A detailed summary of the quality assessment can be found in Table 1.

**TABLE 1:** Summary of quality assessment details and judgment for risk of bias of each study.

Study	Sequence generation		Allocation concealment		Blinding of participants and personnel		Blinding of outcome assessment		Incomplete outcome data	
	**Judgment**	**Comments**	**Judgment**	**Comments**	**Judgment**	**Comments**	**Judgment**	**Comments**	**Judgment**	**Comments**
Dickerson et al., 2014	Unsure	No mention of sequence generation.	Unsure	No mention of allocation concealment for placebo vs. treatment groups.	Low	Study was double-blind, but there was no mention of how this was maintained.	Low	Study was double-blind, but there was no mention of how this was maintained.	Low	Subjects that were excluded were documented with reasoning.
Kazemi et al., 2019	Low	Patients were randomly assigned to experimental groups (1:1:1) in blocks of 6.	Low	Participants, clinicians, and raters remained blind to the allocated group of each participant.	Low	Participants, clinicians, and raters remained blind to the allocated group of each participant.	Low	Participants, clinicians, and raters remained blind to the allocated group of each participant.	Low	Subjects that were excluded were documented with reasoning.
Ghorbani et al., 2018	Unsure	No mention of treatment group sequencing.	Low	Double-blind study with 1:1 ratio for treatment vs. placebo randomization.	Low	Double-blind study. Throughout the study, the psychiatrist, the rater (study researchers), and the patients were all blind to allocation.	Low	The raters (study researchers) were blind to allocation.	Low	Subjects that were excluded were documented with reasoning.
Eskandarzadeh et al., 2021	Low	Patients were randomly assigned using a random numbers table to either treatment or placebo groups.	Low	Patients were randomly assigned using a random numbers table to either treatment or placebo groups.	Low	The study is double-blind, but there was no mention as to how that was maintained.	Low	The study is double-blind, but there was no mention as to if raters were blinded.	Low	Subjects who were excluded were documented with reasoning.
Arifdjanova et al., 2021	Low	Participants were randomized to either experimental or control group, but no mention as to how.	High	No concealment of treatment group.	High	No mention of blinding of staff.	High	No mention of blinding of raters.	Low	Some subjects were not included at the beginning, but criteria for which they were not included was not disclosed.
Miyaoka et al., 2018	Unsure	No mention of allocation sequence.	Unsure	No mention of allocation concealment.	High	No mention of blinding.	High	No mention of blinding.	Low	All data was reported and subjects that were excluded were documented with reasoning.
Rudzki et al., 2019	Low	Patients were randomly assigned to placebo or probiotic group using computer generated randomization list.	Low	The study was blinded at group allocator, participant, and assessor levels.	Low	The study was blinded at group allocator, participant, and assessor levels.	Low	The study was blinded at group allocator, participants, and assessor levels.	Low	Subjects that were excluded were documented with reasoning.
Yang et al., 2021	Unsure	No mention of how the participants were randomized.	High	The blind method was not used in this study, and the researchers were fully aware of the medication.	High	Researchers were fully unblinded and aware of the medication. No mention of unblinding to participants.	High	The blind method was not used in this study, and the researchers were fully aware of the medication.	Low	Subjects that were excluded were documented with reasoning.

## Results

### Search results

Following the removal of 1072 duplicates, the search yielded 3,957 results. Subsequent abstract screening and full-text screening, according to the search criteria highlighted earlier (shown in Figure 1) resulted in 8 papers with direct relevance to the research question.

### Study characteristics

Our findings can be grouped into studies examining three major categories of psychological disorders. For one, 370 patients across 5 trials were categorized and treated as patients with MDD (Ghorbani et al., 2018; Miyaoka et al., 2018; Kazemi et al., 2019; Rudzki et al., 2019; Arifdjanova et al., 2021). A significant majority of these patients received traditional antidepressant medication in the form of SSRIs. The sole trial examining 48 patients with GAD (Eskandarzadeh et al., 2021) assessed the use of sertraline, a common SSRI, as its psychotropic agent. The remaining two trials examined 132 patients with SZ or schizoaffective disorder (SZA) (Dickerson et al., 2014; Yang et al., 2021), one in which participants received the atypical antipsychotic Olanzapine, and the other in which participants continued taking whichever antipsychotic they were prescribed prior to enrolling in the study. As can be seen in Table 2, the studies were conducted across 6 different countries and on predominantly female populations. Furthermore, while the majority of studies used an SSRI or atypical antipsychotic, the makeup and the quantity of probiotic administered varied greatly across trials, reducing the generalizability of conclusions.

**TABLE 2:** Summary of key study characteristics and outcomes.

References	Study population	Study design	Sample size	Mean age (%F)	Country	Intervention type	Duration	Probiotic	Outcome measures	Conclusion
Arifdjanova et al., 2021	Mild-moderate MDD (ICD-10), 18–45yo	Placebo, double-blind RCT	*n* = 149	32.9 (62.2%)	Russia	Cipralex (SSRI) + Placebo or Probiotic	6 weeks	Bac-Set Forte* 3 capsules/day (10^10^ CFU)	HAM-D for depression severity, ELISA for cortisol and cytokines, HPLC for blood plasma	Found decreased levels of cortisol, dopamine, IL-6, TNF-a and nitric oxide, and a bigger reduction in depressive symptoms in the adjuvant PB group compared to standard therapy.
Rudzki et al., 2019	Moderate MDD (DSM-IV-R)	Placebo, double-blind RCT	*n* = 60	39 (71%)	Poland	Antidepressant (various SSRIs) + Probiotic or Placebo	8 weeks	2 capsules/day (10 × 10^9^ CFU of Lactobacillus Plantarum 299v each)	Symptom Severity: HAM-D 17, SCL-90, PSS-10, cognitive function, biochemical parameters also assessed	PB correlated with increased cognitive performance and decreased kynurenine concentration in MDD patients, no significant effect on symptom severity.
Ghorbani et al., 2018	Moderate MDD (DSM-V), 18–55yo	Placebo, double-blind RCT	*n* = 40	34.8 (70%)	Iran	Fluoxetine (SSRI, 20 mg/day - 4W) then Fluoxetine + synbiotic capsule or Placebo (6W)	6 weeks	1 capsule/day (MS probiotic**, 500 mg + prebiotic, 100 mg)	HAM-D primary outcome	Found a greater reduction in HAM-D scores in synbiotic treated patients compared to the placebo group.
Kazemi et al., 2019	Mild-moderate MDD (ICD-10) on medication, 18–50yo	Three-arm placebo, double-blind RCT	*n* = 81	36.5 (70.9%)	Iran	Antidepressant (sertraline, fluoxetine, citalopram, amitriptyline) + Probiotic or Prebiotic or Placebo	8 weeks	1 sachet/day - probiotic (≥10 × 10^9^ CFU Lactobacillus helveticus and Bifidobacterium longum), or prebiotic (galactooligosaccharide)	BDI primary outcome, HPLC for serum tryptophan and branched chain amino acids, ELISA for kynurenine	PB resulted in a decrease in BDI score and increased tryptophan/isoleucine ratio compared to placebo and prebiotic. No significant results for prebiotic and placebo groups
Miyaoka et al., 2018	Treatment Resistant MDD (DSM-IV)	Prospective open label randomized	*n* = 40	43.5, (60%)	Japan	Antidepressant (fluvoxamine, paroxetine, escitalopram, duloxetine, and sertraline) with or without (control) Probiotic	8 weeks	60 mg/day (Clostridium butyricum MIYAIRI (CBM588)–10 CFU/gram)	HAM-D, BDI and the Beck Anxiety Inventory	PB correlated to significant improvement in depression regardless of antidepressant type; well tolerated.
Eskandarzadeh et al., 2021	Drug-free patients with GAD (DSM-V), 18–65yo	Placebo, double-blind RCT	*n* = 48	33.9 (81.2%)	Iran	Sertraline (SSRI, 25 mg/day) + Placebo or Probiotic	8 weeks	1 capsule/day (18*10^9^ CFU Bifidobacterium longum, Bifidobacterium bifidum, Bifidobacterium lactis and Lactobacillus acidophilus)	HAM-A scale for anxiety, Beck Anxiety Inventory, State-Trait Anxiety Inventory	Found Sertraline + PB group to have improved clinical outcome measures as opposed to Sertraline + placebo. Significance varied depending on scale used.
Dickerson et al., 2014	Mild-moderate SZ (DSM-IV, PANSS), 18–65yo	Placebo, double-blind RCT	*n* = 65	46.2 (35.4%)	U.S.	Antipsychotic (various) + Placebo or Probiotic	14 weeks	1 Capsule/day (10^9^ CFU combined Lactobacillus rhamnosus strain and Bifidobacterium animalis subsp. lactis strain Bb12)	PANSS to measure psychiatric symptoms + difficulty of bowel movement scale	No significant difference in psychiatric scores, PB well tolerated, PB group had less bowel problems associated w/treatment.
Yang et al., 2021	First-episode SZ or SZA (DSM-V), 18–55yo	Open-label, RCT	*n* = 67	43.2 (67.7%)	China	Olanzapine with or without (control) Bifidobacterium group	12 weeks	3 capsules/day (live combined Bifidobacterium, Lactobacillus, and Enterococcus capsules; 1 × 10^9^ CFU each)	Body weight, BMI, appetite, latency to increased appetite, and baseline weight increase of more than 7%, PANSS to measure psychiatric symptoms	No significant differences in PANSS scores. In the first 4 weeks there was reduced weight change and BMI for PB group, but this difference disappeared after 4 weeks. There were no overall differences in appetite.

*Contents of Bac-Set Forte: *Streptococcus thermophilus*; *Bifidobacterium* ssp; *Lactobacillus* ssp. among others. **Contents of MS probiotic: *L. casei* = 3 × 10^8^, *L. acidophilus* = 2 × 10^8^, *L. bulgaricus* = 2 × 10^9^, *L. rhamnosus* = 3 × 10^8^, *B. breve* = 2 × 10^8^, *B. longum* = 1 × 10^9^, *S. thermophilus* = 3 × 10^8^.

In all studies, a subset of patients received their psychotropic medication in conjunction with a type of probiotic supplementation. Most of these studies were conducted in the form of a double-blinded, randomized clinical trial. Two studies, however, (Miyaoka et al., 2018; Yang et al., 2021) lacked a placebo arm, following an open-label randomized format.

### Efficacy of probiotics as an adjuvant therapy

In the eight studies examined (shown in Table 2), the medications to treat psychiatric illness were either antidepressants (*n* = 6), or antipsychotics (*n* = 2). All studies included measures for clinical outcomes and symptom severity. In the depression studies, the majority used the Hamilton Depression Rating Scale (Hamilton, 1960) (HAM-D) (*n* = 4) or the Beck Depression Inventory (Beck et al., 1996) (BDI) (*n* = 2) to measure symptoms of mood, anhedonia, sleep, anxiety, appetite, and other symptoms associated with depression. The anxiety study used the Hamilton Anxiety Rating Scale (Hamilton, 1959) (HAM-A) (*n* = 1) to assess symptoms of anxiety such as mood, tension, insomnia, physiological symptoms. The schizophrenia studies used the Positive and Negative Syndrome Scale (Kay et al., 1987) (PANSS) (*n* = 2) to assess positive and negative symptoms associated with schizophrenia such as delusions, hallucinations, blunted affect, and social withdrawal. Higher scores on these scales correspond to an increased severity of the illness.

When examining the effects on symptom severity in patients with MDD or GAD, five of the six studies found that patients who received adjuvant probiotic treatment had significant reductions in symptom severity on the majority of the scales used. One study (Rudzki et al., 2019) did not find any significant effects on symptom severity in patients with MDD receiving adjuvant probiotic therapy using the HAM-D, Symptom Checklist-90 (Derogatis and Savitz, 1999) (SCL-90), and Perceived Stress Scale-10 (Cohen et al., 1983) (PSS-10), but did find that adjuvant probiotic treatment was correlated with increased cognitive performance. Neither of the two studies using patients with schizophrenia or schizoaffective disorder taking antipsychotics found adjuvant probiotic treatment to have any effect on the psychiatric symptoms. That being said, both studies found adjuvant probiotic treatment to reduce the adverse events and side effects associated with antipsychotic treatment, with Dickerson et al. finding fewer reports of bowel difficulties, and Yang et al. finding a reduced weight gain in the first 4 weeks in the adjuvant probiotic groups. Though the reduced weight gain in the study conducted by Yang et al. was transient, with no differences between the adjuvant and monotherapy groups by weeks 8 and 12, adjuvant probiotic therapy was found to eliminate the observed sex-based differences in weight gain seen in the olanzapine monotherapy group. There was no significant difference in body weight change between men and women in the adjuvant probiotic group, whereas there were significantly higher increases in the body weight of women compared to men in the olanzapine monotherapy group.

## Discussion

The clinical outcome findings from the studies included in this review suggest probiotic and synbiotic adjuvant treatment with SSRIs for MDD and GAD to be more effective in decreasing depressive and anxious symptomology, respectively, than SSRI treatment alone. In the one study included in this review that used included a prebiotic group, prebiotics alone were not found to have a significant effect on clinical symptoms. The improved clinical outcomes of probiotic adjuvant treatment for MDD and GAD were found to be persistent throughout the course of the treatment, but further long-term follow-up assessments would be needed to investigate the persistence of this effect when treatment is discontinued. For individuals with schizophrenia, adjuvant probiotic treatment was not found to be more effective in reducing clinical symptom severity than standard antipsychotic treatment alone. Some potential limitations that could explain this lack of effect on clinical symptoms and outcomes are discussed in the conclusion. Though there was no significance in clinical findings for the schizophrenia groups, adjuvant probiotic treatment was associated with a decrease in treatment associated adverse events and side effects. The findings of these studies suggest adjuvant probiotic treatment to have an alleviative effect on some of the gastrointestinal adverse events associated with antipsychotic treatment, such as weight gain and bowel problems. Some of these beneficial effects were found to be fairly long lasting, as is the case in the trial by Dickerson et al. (2014), but some effects were found to be transient, with Yang et al. finding a reduced weight gain for only the first 4 weeks of adjuvant probiotic treatment (Yang et al., 2021).

As mentioned in the introduction, some antipsychotics and antidepressants have been found to have antibacterial properties, as such, it is important to consider the specific medications used in each study. All MDD and GAD studies used participants taking SSRIs, with Miyoaka et al. including participants taking the SNRIs duloxetine (*n* = 9) and milnacipran (*n* = 3) and Kazemi et al. including participants taking the tricyclic antidepressant amitriptyline (n not reported). The SSRIs involved in the studies included escitalopram, citalopram, fluoxetine, fluvoxamine, paroxetine, and sertraline. It has been found SSRIs vary in the degree to which the inhibit bacterial growth, with sertraline and fluoxetine having the strongest antimicrobial activity, followed by paroxetine and fluvoxamine, and then escitalopram and citalopram (McGovern et al., 2019). Amitriptyline has also been found to have antimicrobial effects to around the same degree as paroxetine (Mandal et al., 2010). The antimicrobial effect of SNRIs is less clear, with studies finding venlafaxine to have no effect (Ait Chait et al., 2020), and others finding the clinical effects of duloxetine to be reduced by the bacteria Ruminococcus flavefaciens (Lukić et al., 2019), suggesting an interaction between duloxetine and the bacteria. Despite the differences in medications used and their degree of antimicrobial activity, all the MDD and GAD studies found results suggesting probiotics combined with antidepressants improved clinical outcomes when compared to antidepressants alone.

For the schizophrenia studies, all participants in the study conducted by Yang et al. took olanzapine, whereas participants in the Dickerson et al. study took a variety of antipsychotics. The antipsychotics used in the Dickerson et al. study were clozapine (*n* = 17), olanzapine (*n* = 15), risperidone (*n* = 15), aripiprazole (*n* = 11), quetiapine (*n* = 9), haloperidol (*n* = 7), ziprasidone (*n* = 5), and asenapine (*n* = 1), including some participants that took more than one antipsychotic. Though a variety of antipsychotics were used in the Dickerson et al. study, all fall under the category of atypical antipsychotics except for haloperidol which is a butyrophenone derivative. Olanzapine has been found to shift the fecal microbiota in mice toward an “obesogenic” profile (Morgan et al., 2014), and all of the atypical antipsychotics used in the Dickerson et al. study have been found to be associated with significant changes in the gut microbiome and a decrease in species diversity in females (Flowers et al., 2017). Haloperidol has also been found to have some bacteria inhibiting effects (Korbee et al., 2018). Taking that into consideration, though the two studies used different antipsychotics, they both used primarily atypical antipsychotics, but the exact degree in which each individual antipsychotic effects the gut microbiome is not certain. As such, it is possible that the use of differing antipsychotics could have contributed to the difference in longevity of the observed beneficial effects.

The exact mechanisms of action for these beneficial effects of probiotic adjuvant treatment are not fully understood. Though the exact pathway and importance of the various pathways by which probiotic adjuvant treatment may exert its effect is not known, multiple pathways have been described for ways in which the microbiome affects the brain and central nervous system. Biomarker data collected in the MDD studies suggests that the probiotic adjuvant treatment exerts its therapeutic effect through effects on the immune system, the hypothalamic pituitary adrenal axis (HPA-axis), and the tryptophan system. Decreased concentrations of immune markers such as interleukin-6, tumor necrosis factor-a, and nitric oxide suggests a decrease in the activity of the immune system in response to probiotic adjuvant treatment. The immune system has long been known to be intimately linked to MDD and depressive symptomology, with an over-active immune system often being observed in individuals with MDD (Leonard, 2010). One proposed mechanism of action for microbiome targeting treatment for MDD is a reduction in immune system activity through a decrease in gut permeability. It is thought that individuals with MDD and/or other illnesses have increased gastrointestinal permeability, allowing for microorganisms and other potentially harmful toxins from the gut to pass into the body, resulting in an increase in inflammation and immune system activity. Repopulation of the gut microbiome and/or the introduction of beneficial bacteria through probiotic treatment is thought to alleviate this increased gut permeability, and thus reduce inflammation and immune system activity, leading to a decrease in depressive symptomology. Biomarker data from the study conducted by Arifdjanova et al. (2021) also found cortisol levels to be decreased in the probiotic adjuvant treatment group. Cortisol is often associated with stress and is a major indicator HPA-axis activity. Individuals with MDD and/or GAD often are found to have an overactive HPA-axis and increased levels of cortisol. Probiotic and other gut microbiome targeting treatments have been frequently found to have an inhibitory effect on HPA-axis activity and has been associated with decreased cortisol levels. Influencing the HPA-axis and cortisol production and availability seems to be another pathway by which probiotic adjuvant treatment results in improved clinical outcomes. Another potential mechanism for the improved clinical outcome observed in the probiotic adjuvant treatment groups is through effects on the tryptophan system. Kazemi et al. (2019) and Rudzki et al. (2019) found an increased tryptophan/isoleucine ratio and decreased kynurenine (a tryptophan metabolite) concentration in the probiotic treatment groups of their respective studies. Tryptophan is a precursor to serotonin, a neurotransmitter that has long been associated with MDD and GAD. SSRIs exert their therapeutic action by inhibiting serotonin reuptake transporter proteins, leading to an increase in the relative abundance and concentration of serotonin in the brain. Though serotonin is often associated with the brain and psychiatric illnesses, up to 90% of the body’s serotonin is produced in the gut. The mechanism by which the gut microbiome affects serotonin production and availability is thought to be through interactions with microbiome metabolites and enterochromaffin cells (EC cells) in the gut. The microbiome produces long and short chain fatty acids (SCFA) through the fermentation of non-digestible carbohydrates. Long chain fatty acids influence serotonin production indirectly through interactions with glucagon-like protein-1 (GLP-1) cells leading to increased GLP-1 which interacts with EC cells to increase serotonin production and availability. Short chain fatty acids interact directly with EC cells to increase serotonin production and availability. In addition to these interactions with EC cells, short chain fatty acids have the ability to cross the gut-blood and blood-brain barriers, and are thought to have an anti-inflammatory effect, thus further influencing the immune system. The exact bacteria involved in these processes are not fully characterized, but some species *Lactobacillus* and *Bifidobacterium* have been found to produce neurotransmitters such as acetylcholine and gamma-aminobutyric acid, and *Streptococcus*, *Enterococcus*, and *Escherichia* have been found to produce serotonin, dopamine, and epinephrine (Galland, 2014). These biomarker findings suggest that the beneficial clinical outcomes in the adjuvant probiotic groups with MDD and GAD are a result of the adjuvant probiotic treatment affecting multiple if not all of the pathways by which the microbiome and brain are connected.

As for the observed effect of a decrease in gastrointestinal adverse events in the adjuvant probiotic group of the schizophrenia studies, the mechanism of action is largely unclear. The alleviation of gastrointestinal issues, such as constipation, most likely acted through interactions with the serotonin pathway. As described above, probiotic administration may have resulted in an increase in SCFAs produced by the microbiome, which in turn increases serotonin synthesis through EC cells. Serotonin has been found to activate enteric neural circuitry to initiate peristalsis and reduce constipation (Crowell, 2004). The transient effect of decreasing weight gain for the first 4 weeks could be from a variety of factors. Bifidobacterium administration has been linked to both weight gain and weight loss depending on the strain (Yin et al., 2010). The exact strain used by Yang et al. was not reported (Miyaoka et al., 2018), but increased bacteria with bile salt hydrolase has been found to prevent weight gain through the deconjugation of bile acids (Joyce et al., 2014). This may have been the case for the Yang et al. study, but the general negative impact on energy by olanzapine as well as its own mechanism of action for weight gain may have outweighed the preventative action of the probiotic over time. Although it is thought that probiotics may improve clinical outcomes and symptom severity in populations with schizophrenia through interaction with the immune system (Fond et al., 2020), neither of the studies included a robust collection of immune system related biomarkers, and thus the effect of adjuvant probiotic treatment on the immune system of the patients in these studies is unknown.

## Conclusion

Although the studies included in this review were generally found to be of high quality with low risk of bias, there were still some limitations to these studies that impact the generalizability and conclusions that could be drawn from their findings. A major limitation is the small number of studies for each psychiatric illness and the lack of studies investigating other psychiatric illnesses. With only eight studies in total, five of which used a population with MDD, it is difficult to draw strong generalizable conclusions about the effect of adjuvant probiotic treatment on GAD and schizophrenia. Additionally, though the studies included had relatively large sample sizes, further larger scale, double blind, randomized controlled trials are required in the future to make any definitive conclusions. Many of the studies included in this review also did not have comprehensive biomarker collection. To be able to elucidate the mechanisms of action of probiotic supplementation as an adjuvant treatment, as well as to evaluate the colonization of the gut by the probiotics administered and changes in key features of the gut such as intestinal permeability, robust and consistent biomarker collection is necessary. This collection would include biomarkers for the immune system, HPA-axis, serotonin system, and the gut microbiome. Another limitation of the studies was the fact that many used a variety of first-line antidepressant or antipsychotic treatments in combination with the probiotics, which is helpful for evaluating adjunctive probiotic treatment in general but does not give strong insight into the effectiveness of adjunctive probiotic administration with specific antidepressants and antipsychotics. As different psychiatric treatments can have differing effects on the gut microbiome, studies or analyses focusing on one specific intervention could allow for more detail in determining what combination of treatment would be most effective for an individual. These studies are also limited by their length, with the majority being unable to have significant long-term follow-ups to investigate the longevity of the observed effects. Another limitation in this field is the lack of consensus on dosages for probiotics as well as the treatments they are given in combination with. Dose finding studies in the future are needed, in addition to studies investigating the efficacy of adjuvant probiotic treatment at different stages and severity of the illnesses. This is especially relevant for the schizophrenia studies, which included populations with relatively severe clinical symptoms and later stages of the illness. It is possible that in a population with milder symptoms and a more recent onset, adjuvant probiotic therapy could effectively impact clinical outcomes.

Despite these limitations, the findings of these studies suggest the use of adjuvant probiotic treatment with SSRI treatment for MDD and GAD to be superior to SSRI treatment alone. Probiotic adjuvant treatment with antipsychotics could be beneficial for improving the GI issues associated with antipsychotics, but these findings do not suggest that adjuvant probiotic treatment would result in improved clinical outcomes for symptoms of schizophrenia. Though progress in psychiatric research is challenging, these studies have shown that combining probiotic treatment with first line pharmaceutical treatments is promising, and their findings certainly justify continued research in this area. The gut microbiome and the brain are clearly linked, and these studies show that combining treatments that target both areas, respectively, is a viable and efficacious way to combat the symptoms and treat psychiatric illnesses.

## Data availability statement

The original contributions presented in this study are included in this article/supplementary material, further inquiries can be directed to the corresponding author.

## Author contributions

BB and AS completed the initial search of the databases, adhering to the search strategy. EF and one of BB or AS independently assessed the titles and abstracts of records retrieved from a systematic search according to the identified inclusion and exclusion criteria. BB and EF completed the full-text review. AC resolved any disagreements. CS completed quality assessment of eligible articles. All authors contributed to the article and approved the submitted version.
